# Vibrational Signatures of Unrealized Phosphorus Suboxide Intermediates in White Phosphorus Oxidation Reactions

**DOI:** 10.1021/acs.jpca.5c06891

**Published:** 2025-12-26

**Authors:** Ethan J. Poncelet, Mitchell E. Lahm, Anna G. Poncelet, Justin M. Turney, Michael A. Duncan, Henry F. Schaefer, Yohannes Abate

**Affiliations:** † Center for Computational Quantum Chemistry, 1166 University of Georgia, Athens, Georgia 30602, United States; ‡ Department of Chemistry, 1166 University of Georgia, Athens, Georgia 30602, United States; § Department of Physics and Astronomy, 1166 University of Georgia, Athens, Georgia 30602, United States

## Abstract

White phosphorus ignition notoriously produces the phosphoric acid anhydride P_4_O_10_, yet the intermediate oxidation steps remain undetermined. We report the first geometric and vibrational characterization of two P_4_O_2_ isomers, P_3_OPO and P_3_PO_2_, and substantiate a previously proposed cyclic P_4_O_2_ isomer. We formally assign the infrared bands observed by Andrews and Mielke at 898 and 891 cm^–1^ to the antisymmetric P–O–P vibrations of P_3_OPO species (Mielke and Andrews, 1990). Additional bands corresponding to terminal P=O and −PO_2_ stretches of P_3_OPO and P_3_PO_2_ discussed herein also went unrecognized due to the peculiar bonding of P_4_O_2_ species compared to oxo-bridged P_4_O_*x*_ (*x* = 3–6) species. Sequential addition of oxygen atoms to the P_4_ tetrahedron appears to form P_3_OPO and P_3_PO_2_, while cyclic P_4_O_2_ is formed from P_2_O dimerization. CCSD­(T) geometries, CCSD­(T) + MP2­[δVPT2] fundamental frequencies, and enthalpies of formation extrapolated using focal-point analysis are reported. The predicted enthalpies of formation relative to tetrahedral P_4_ plus ^3^Σ_*g*_^–^ O_2_ for *bent*-P_3_OPO, *extended*-P_3_OPO, P_3_PO_2_, and *cyclic*-P_4_O_2_ are −93, −88, −85, and −63 kcal mol^–1^, respectively.

## Graphical abstract

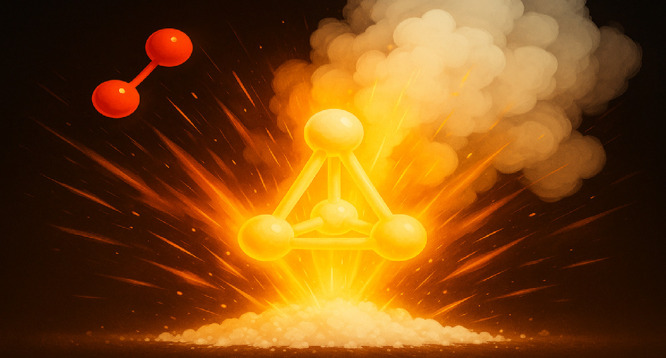

## 1 Introduction

In an attempt to find the Philosopher’s Stone, alchemist Hennig Brand boiled 1200 gallons of urine from himself, his family, and local pubgoers until he was left with a glowing substance.^2^ Brand had synthesized white phosphorus (P_4_), which ignites and produces an intense chemiluminescence upon contact with oxygen. The adamantane-backbone analogue P_4_O_6_ and phosphoric acid anhydride P_4_O_10_ are iconic products of white phosphorus ignition,^3–13^ yet intermediate species involved in their formation remain undetermined. Chemiluminescence provides a convenient probe into P_4_ oxidation reactions, and PO_2_^*^ is generally considered the predominant continuum emitter.^14–22^ However, most species involved are nonemissive.

Andrews and co-workers were the first to address this limitation by characterizing the lower oxides of phosphorus in a series of matrix-isolation IR experiments.^1,23–28^ They identified PO, PO_2_, PO_2_^–^, bridged (B-)­P_4_O, terminal (T-)­P_4_O, and bands that suggested a series of P_4_O_*x*_ (*x* = 2–5) species from reactions of P_4_ with oxygen atoms from O_2_ and O_3_ in argon.^1^ UV–vis irradiation was required for reactions of P_4_ and O_3_ to proceed. Higher O_3_ concentrations, shorter wavelengths of irradiation, and longer irradiation times appeared to encourage the formation of P_4_O_*x*_ (*x* = 2–5) species.^1,23^ Reactions of oxygen atoms from microwave discharge of O_2_ with P_4_ were spontaneous, and produced the aforementioned P_4_ + O_3_ products as well as P_2_O and cyclic (C−)­P_4_O.^23^ Experiments codepositing P_2_ with O_3_ have formed these additional products,^25,26^ suggesting the microwave discharge caused substantial P_4_ dissociation before reacting with oxygen atoms.

Gas-phase IR and visible emission spectra of PO, PO_2_, P_2_, and P_2_O from reactions of P_4_ vapor with O_2_ and oxygen atoms in helium were collected by Hamilton and co-workers.^14^ PO absorption was maximized under low O_2_ concentrations while PO_2_ absorption was maximized under high concentrations, suggesting PO is required for PO_2_ formation. Both Hamilton and Andrews concluded P_4_ + O → PO + P_3_ must be a dominant initial reaction pathway of P_4_. Simultaneous maximization of P_2_^*^ emission with the mass 78 signal of P_2_O suggested P_4_ + O → P_2_O + P_2_ was also an initial reaction pathway, which was predicted but not observed by Andrews and co-workers.^1,23^

Raman and IR absorption spectra of bulk phosphorus materials have been reported.^29–34^ Stability of these materials in ambient conditions is a concern, and theoretical oxidation and hydrolysis mechanisms have been proposed.^35–37^ Nano-FTIR spectra of black and violet phosphorus flakes have resolved −PO_2_, terminal P=O, and bridged P–O–P vibrational peaks, giving rise to the first structural changes observed in situ.^32^ To our knowledge, nano-FTIR has not been used to observe surface oxidation of the more-volatile, white allotrope of phosphorus.

Given that P_2_O formation and sequential oxygen addition to the P_4_ tetrahedron are evidenced by previous experiments, we investigated the role of P_4_O_2_ species in white phosphorus oxidation pathways. Two theoretical studies precede our work. Peyerimhoff and co-workers reported qualitative structures and energies of low-lying P_4_O_2_ species using B3LYP/DZP, but did not compute vibrational frequencies.^38^ Yao and co-workers reported two P_4_ + O_2_ → P_4_O_2_ reaction pathways using B3LYP/6–311++G**, a peculiar basis set for the system, and experimental bands associated with the computed fundamental vibrational frequencies of their P_4_O_2_ species remain undetected.^39^ We present geometries, energies, and vibrational frequencies of three distinct P_4_O_2_ isomers present in white phosphorus oxidation reactions. These predictions are related to previous experimental work.^1,14,23–28^

## 2 Methods

After unsuccessfully determining a molecular P_4_ + O_2_ → P_4_O_2_ reaction pathway that produces a P_4_O_2_ species with vibrational modes observed by Andrews and co-workers,^1,25,26^ the ABCluster program^40,41^ was used to generate 5000 candidate minima along the P_4_O_2_ potential energy surface (PES) with the semiempirical extended tight-binding (xTB)^42–44^ method and artificial bee colony algorithm. Duplicate structures were removed using total and nuclear repulsion energies. Energies of the remaining structures were computed using B3LYP-D3BJ/6–311+G*,^45–48^ and the lowest-lying structures were then optimized at the same level of theory. Orca 6.0 ^49–53^ was used for all DFT computations.

Optimizations and harmonic vibrational frequency computations at the MP2 and coupled cluster singles, doubles, and perturbative triples [CCSD­(T)][Bibr ref54] levels of theory with the cc-pV­(T+d)­Z basis set^55–60^ were performed on four P_4_O_2_ structures, where the additional tight d-type polarization function is only added to phosphorus atoms. MP2/cc-pV­(T+d)­Z second-order vibrational perturbation theory (VPT2)^61,62^ was used to account for anharmonicity. Fundamentals reported are thus harmonic CCSD­(T) plus MP2­[δVPT2] anharmonic composite vibrational frequencies. Harmonic MP2/cc-pV­(T+d)­Z ^18^O frequencies were also determined, and shifts are reported as a ratio of (ν_MP2_^^18^ O^/ν_MP2_^^16^ O^) with our fundamental frequency. Similar composite schemes targeting reliability at reduced computational costs have been shown to be effective.^63–68^ Single-point energy computations were conducted with HF, MP2, CCSD, and CCSD­(T) with cc-pV­(n+d)­Z (n = D, T, Q) basis sets. DLPNO–CCSDT^69,70^ and -(Q)^71,72^ energies with the cc-pV­(D+d)­Z basis set were also computed. Focal-point analysis^73–76^ was used to extrapolate these energies to near the complete basis set (CBS) limit, and harmonic and anharmonic zero-point vibrational energy contributions are included. Frozen cores with the electron configurations of helium and neon were used for oxygen and phosphorus, respectively. CFOUR 2.0 ^77^ was used for HF, MP2, CCSD, and CCSD­(T) computations, except for the use of MOLPRO 2022.1 ^78–80^ for the single-point energy computations in Figure 2. DLPNO–CCSD­(T),^81^ -T,^70^ and -(Q)^72^ code was provided by Andy Jiang and co-workers utilizing Psi4 1.4.^82^ NBO 7.0 ^83^ was used to investigate natural bonding orbitals also utilizing Psi4 1.4.

**Figure 1:**
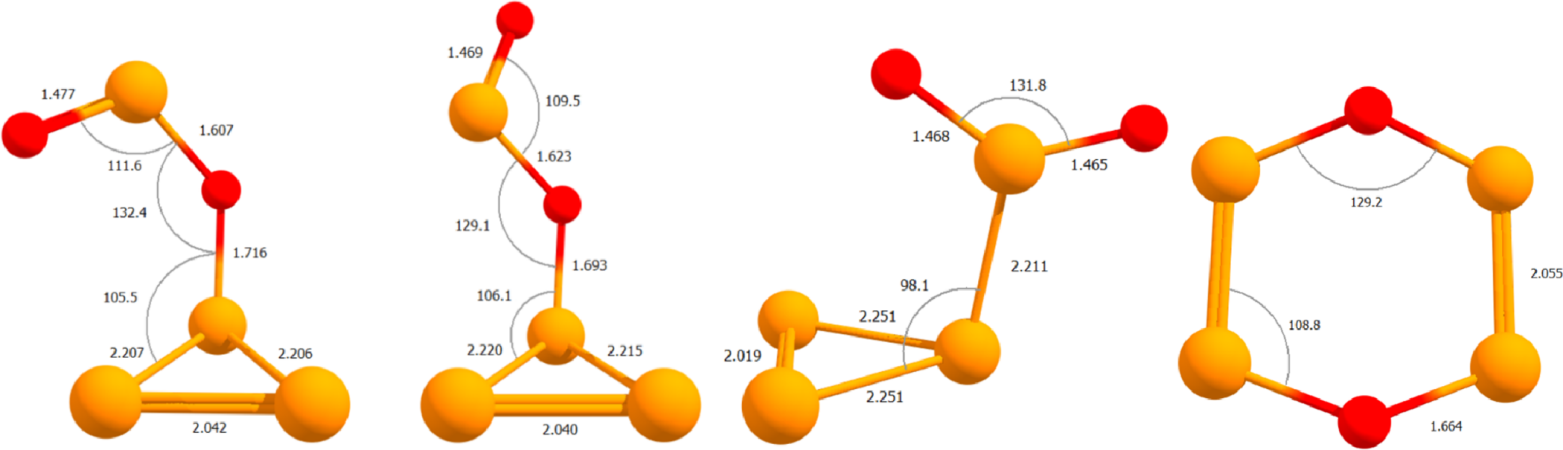
From left to right, geometries of bent-P_3_OPO, extended-P_3_OPO, P_3_PO_2_ (C_s_), and cyclic P_4_O_2_ (C_2*v*_) are shown.

## 3 Results and Discussion

### 3.1 Geometries

Hamilton and co-workers obtained rotationally resolved spectra for P_2_O, with terminal P=O stretch bands yielding a *B*_0_ + *B*_1_ of 0.255 cm^–1^ in their gas-phase P_4_ + O in helium experiment.^14^ We calculated a 2*B* value of 0.257 cm^–1^ for P_2_O from a CCSD­(T)/cc-pV­(T+d)­Z geometry optimization (*r*_*P*–*P*_ = 1.903; *r*_*P*–*O*_ = 1.476 Å), which gives us confidence in the method for predicting structures of other phosphorus suboxides. Geometries of *bent*-P_3_OPO (*C*_1_), *extended*-P_3_OPO (*C*_1_), P_3_PO_2_ (*C*_*s*_), and cyclic P_4_O_2_ (*C*_2*v*_) at the CCSD­(T)/cc-pV­(T+d)­Z level of theory are presented in Figure 1. The P_3_OPO rotamers and P_3_PO_2_ isomer feature P_3_ near-isosceles triangles bound to a PO_2_ motif. In the P_3_OPO rotamers, a single P–O bond connects the P_3_ and PO_2_ units; whereas a single P–P bond connects the P_3_ and PO_2_ units in P_3_PO_2_. All three contain a P=P double bond at the base of the P_3_ triangle, with single P–P bonds connecting the base to the apex. Terminal oxygen atoms in the P_3_OPO rotamers are doubly bound to the phosphorus in the PO_2_ units. The P_3_PO_2_ isomer features two terminal oxygen atoms, forming a delocalized PO_2_ motif with P–O bond orders of 1.5. Containing none of the aforementioned features, the cyclic boat P_4_O_2_ isomer has double P=P bonds and single P–O bonds.

Hewitt and co-workers conducted gas-phase electron diffraction studies by of P_4_O_6_ and P_4_O_10_, which revealed respective bridged P–O bond lengths of 1.638 and 1.604 Å, a terminal P=O length of 1.429 Å, and P–O–P angles of 123.5 and 126.4°.^3,5^ Terminal P=O bond lengths between 1.465 and 1.477 Å were computed for our P_4_O_2_ species, which are slightly longer than found in P_4_O_10_ but compare nicely to the 1.470 Å P–O bond in T-P_4_O computed by Lohr and co-workers.^84^ The P_3_OPO rotamers have P_3_–OPO bond lengths of 1.716 and 1.693 Å, which are much longer than their P_3_O–PO bond lengths of 1.607 and 1.623 Å. This behavior is expected considering the structures of P_4_O_6_ and P_4_O_10_, where bridged P–O bonds are shorter when adjacent to a terminal oxygen. Double P=P bond lengths range from 2.019 Å in the P_3_PO_2_ structure to 2.055 Å in the cyclic boat P_4_O_2_ structure. Single P–P bond lengths range from 2.206 in *bent*-P_3_OPO to 2.251 Å in the P_3_PO_2_ structure.

P–O–P angles across all four structures range from 129.1° in *extended*-P_3_OPO to 132.4° in *bent*-P_3_OPO. Andrews and co-workers suggested a P–O–P angle of 127° for the cyclic boat P_4_O_2_ structure based on isotopic shifts and the three atom G-matrix element.^26^ We compute an angle of 129.2°. O–P–O angles are much smaller in the PO_2_ units of the P_3_OPO rotamers (111.6 and 109.5°) than the O–P–O angle in the PO_2_ motif of the P_3_PO_2_ isomer (131.8°). A lone pair on the phosphorus atom of the PO_2_ unit in the P_3_OPO rotamers reduces the O–P–O angle compared to the P_3_PO_2_ isomer, of which the phosphorus in its PO_2_ motif has no lone pair.

### 3.2 Vibrational Frequencies

Fundamental vibrational frequencies, IR intensities, ^18^O shifts, and descriptions of vibrational modes for the four P_4_O_2_ species are reported in Tables 1–4. Previously observed experimental bands are in parentheses next to our computed values. Andrews and Mielke tentatively assigned two bands, 898 and 891 cm^–1^, to antisymmetric P–O–P vibrations of P_4_O_2_ species.^1^ These bands fell between the B–P_4_O (856 cm^–1^) and P_4_O_3_ (916 cm^–1^) antisymmetric P–O–P bands, which led to the assignment. Our computed antisymmetric P–O–P vibrational modes of *bent*- and *extended*-P_3_OPO (Tables 1 and 2) have frequencies of 867 and 853 cm^–1^, and large intensities of 568 and 698 km mol^–1^. These bands were red-shifted by 36 cm^–1^ in ^18^O experiments, and our calculated respective shifts are 35 and 38 cm^–1^. Therefore, we conclude P_3_OPO is responsible for the 898 and 891 cm^–1^ bands tentatively assigned to P_4_O_2_,^1^ not a chemically intuitive, doubly oxo-bridged structure.^38,39^ In a gas-phase experiment, these P_3_OPO bands should be found between 900 and 920 cm^–1^, as argon matrices tend to redshift vibrational frequencies ca. 10–20 cm^–1^.^14,85^

**Table 1:** Composite Fundamental Vibrational Frequencies and Isotopic Shifts in cm^–1^ and Harmonic IR Intensities in km mol^–1^ for *Bent*-P_3_OPO

*bent*-P_3_OPO (*C*_1_)				
^16^O freq	int.	^18^O freq	sym.	description
45	2	44	a	P_3_–OPO twist
115	3	112	a	P_3_–OPO scissor
128	5	126	a	P_3_–OPO scissor
205	4	200	a	P–P–O bend
266	8	256	a	P–P–O bend
417	2	415	a	antisym. P–P(apex)–P
450	2	446	a	sym. P–P(apex)–P
489	8	480	a	O–P–O scissor
561 (592)^a^	87	544 (570)^a^	a	sym. P–O–P
630	10	630	a	base P–P
867 (898)^a^	568	832 (862)^a^	a	antisym. P–O–P
1234 (1260)^a^	49	1188 (1215)^a^	a	terminal P=O

a Experiment, Andrews and Mielke; P_4_ + O_3_ (ref. 1).

**Table 2:** Composite Fundamental Vibrational Frequencies and Isotopic Shifts in cm^–1^ and Harmonic IR Intensities in km mol^–1^ for *Extended*-P_3_OPO

*extended*-P_3_OPO (*C*_1_)				
^16^O freq	int.	^18^O freq	sym.	description
35	3	34	a	P_3_–OPO twist
68	4	65	a	P_3_–OPO scissor
88	1	86	a	P_3_–OPO scissor
211	1	206	a	P–P–O bend
244	2	236	a	P–P–O bend
359 (351)^a^	22	350 (342)^a^	a	O–P–O scissor
412	<1	412	a	antisym. P–(apex)–P
449	7	449	a	sym. P–P(apex)–P
631	2	628	a	base P–P
650	57	633	a	sym. P–O–P
853 (891)^a^	698	815 (855)^a^	a	antisym. P–O–P
1269 (1269)^a^	122	1222 (1221)^a^	a	terminal P=O

a Experiment, Andrews and Mielke; P_4_ + O_3_ (ref. 1).

Although our computed vibrational frequencies are untypically lower than those measured in a matrix,^14,86,87^ previous theoretical work has suggested that upward scaling of select modes of phosphorus oxides may be necessary for theoretical reproduction of experiment.^28,84^ It is possible the species containing a PO_2_ unit obtained a negative charge in the experiments of Andrews and co-workers, as both PO_2_ and PO_2_^–^ are present in ample quantities. Artificial lengthening of bridged P–O bonds or depression of force constants associated with P–O–P vibrational modes could also stem from our methodology. Use of basis sets with higher degrees of polarization and the correlation of core electrons would almost certainly reduce predicted P–O bond lengths, which would in turn raise the computed vibrational frequencies for a better comparison to experiment. Less clear is the effect of higher degrees of polarization of the basis set and correlation of core electrons on the force constants associated with P–O–P vibrational modes, but artificial depression of those force constants could also be the cause of underestimation. We will not discount the possibility that these bands belong to an entirely different molecule; nevertheless, we offer a formal assignment of these P_4_O_2_ vibrational bands after remaining tentatively assigned for multiple decades.^1^

Another band at 1269 cm^–1^ was assigned to a P_4_O_*x*_ terminal P=O vibration, and was shifted to 1221 cm^–1^ in ^18^O experiments.^1^ The *extended*-P_3_OPO terminal P=O vibration was computed here to be 1269 cm^–1^ and has an ^18^O shift of 1222. Unassigned experimental bands at 1411, 1161, 1260, 592, 486, and 351 cm^–1^ were observed to evolve simultaneously under photolysis with the aforementioned P_4_O_2_ bands.^1^ These bands could be, as initially suggested by Andrews and Mielke, adjacent matrix sites containing P_2_ and P_2_O_4_ species causing perturbed P_2_O_4_ vibrations from the polarizable P_2_ molecule. We compute fundamental frequencies of 1409, 1128, 1234, 561, 512, 359, and 356 cm^–1^ for P_3_OPO and P_3_PO_2_, with shifts nearly matching experiment, shown in Tables 1–3.^1,25^ Later work by Bauschlicher and Andrews revealed two OPOPO_2_ conformers (*cis* and *trans*) and O_2_PPO_2_ could be responsible bands in the same regions.^28^ Matrix infrared experiments alone would be insufficient to determine whether these bands belong to P_2_O_4_ or P_4_O_2_ species because both feature terminal and bridged oxygen atoms in addition to a terminal PO_2_ motif. Bauschlicher and Andrews assigned bands at 1473, 1158, and 479 cm^–1^ to O_2_PPO_2_ in their atomic phosphorus + O_2_ in argon experiment, which appear to be distinct from the 1411, 1161, and 486 cm^–1^ bands in the P_4_ + O_3_ in argon experiment. Hence, it is likely that O_2_PPO_2_ bands were detected rather than P_3_OPO and P_3_PO_2_ in the later study with atomic phosphorus. However, P_3_OPO and P_3_PO_2_ species appear to be, at the very least, partially responsible for these other peaks detected in the P_4_ + O_3_ experiments.

**Table 3:** Composite Fundamental Vibrational Frequencies and Isotopic Shifts in cm^–1^ and Harmonic IR Intensities in km mol^–1^ for P_3_PO_2_

P_3_PO_2_ (*C*_*s*_)				
^16^O freq	int.	^18^O freq	sym.	description
35	<1	34	*a* *″*	P_3_–PO_2_ twist
103	2	102	*a* *″*	P_3_–PO_2_ scissor
119	2	115	*a′*	P_3_–PO_2_ scissor
229	1	222	*a′*	P–P–O bend
337	15	332	*a* *″*	PO_2_ wag
356 (351)^a^	27	346 (342)^a^	*a′*	P(apex)–PO_2_
378	5	377	*a* *″*	antisym. P–P(apex)–P
425	6	424	*a′*	sym. P–P(apex)–P
512 (486)^a^	73	503	*a′*	O–P–O scissor
645	<1	645	*a′*	base P–P
1128 (1161)^a^	95	1076 (1117)^a^	*a′*	sym. O–P–O
1409 (1411)^a^	90	1367 (1371)^a^	*a′*	antisym. O–P–O

a Experiment, Andrews and Mielke; P_4_ + O_3_ (ref. 1).

In the P_2_ + O_3_ in argon experiments by Andrews and McCluskey,^25,26^ a band at 867 cm^–1^ was tentatively assigned to the antisymmetric P–O–P vibration of a cyclic P_4_O_2_ molecule. This cyclic P_4_O_2_ species was predicted to be a six-membered ring in a chair or boat conformation with P–O–P angles of 127°. We found no P_4_O_2_ cyclic, chair conformer minimum with comparable P–O–P angles, but did find a six-membered, C_2*v*_ boat P_4_O_2_ minimum. The positive antisymmetric P–O–P combination vibrational frequency was computed to be 893 cm^–1^ with a large intensity of 566 km mol^–1^ (Table 4). An isotopic shift of 38 cm^–1^ was determined here with ^18^O, which is near the experimental shift of 37 cm^–1^. Two peaks at 583 and 506 cm^–1^ match nicely with our theoretical bands of 593 and 506 cm^–1^ for the negative symmetric P–O–P combination and boat “stellation”^88^ vibrational modes of cyclic P_4_O_2_. However, Andrews and McCluskey assigned these features to *trans*-OPOPO_2_ vibrations,^26^ which were later computed by Bauschlicher and Andrews to have high-intensity absorptions right around those peaks as well.^28^ The 583 and 506 cm^–1^ peaks are likely an unresolved combination of *trans*-OPOPO_2_ and cyclic P_4_O_2_ bands. Andrews and McCluskey suggested this structure forms as a result of P_2_O dimerization, and we predict a small barrier of 12 kcal mol^–1^ for 2P_2_O → cyclic P_4_O_2_ (Figure 2).

**Table 4:** Composite Fundamental Vibrational Frequencies and Isotopic Shifts in cm^–1^ and Harmonic IR Intensities in km mol^–1^ for *Cyclic*-P_4_O_2_

*cyclic*-P_4_O_2_ (*C*_2*v*_)				
^16^O freq	int.	^18^O freq	sym.	description
87	1	83	*a*_1_	boat stern bend (+)
146	0	146	*a*_2_	boat twist
214	0	207	*b*_2_	boat stern bend (−)
234	0	227	*a*_1_	boat “rectangulation”^b^
297	0	295	*a*_2_	boat “shear”^b^
470 (506)^a^	38	454	*b*_2_	boat “stellation”^b^
524	4	519	*a*_1_	*P*=P (+)
555	12	554	*b*_1_	P=P (−)
593 (583)^a^	32	582	*b*_2_	sym. P–O–P (−)
654	5	644	*a*_1_	sym. P–O–P (+)
715	0	683	*a*_2_	antisym. P–O–P (−)
893 (867)^a^	566	855 (830)^a^	*b*_1_	antisym. P–O–P (+)

a Experiment, Andrews and McCluskey; P_2_ + O_3_ (ref. 26).

b Ref. 88.

**Figure 2:**
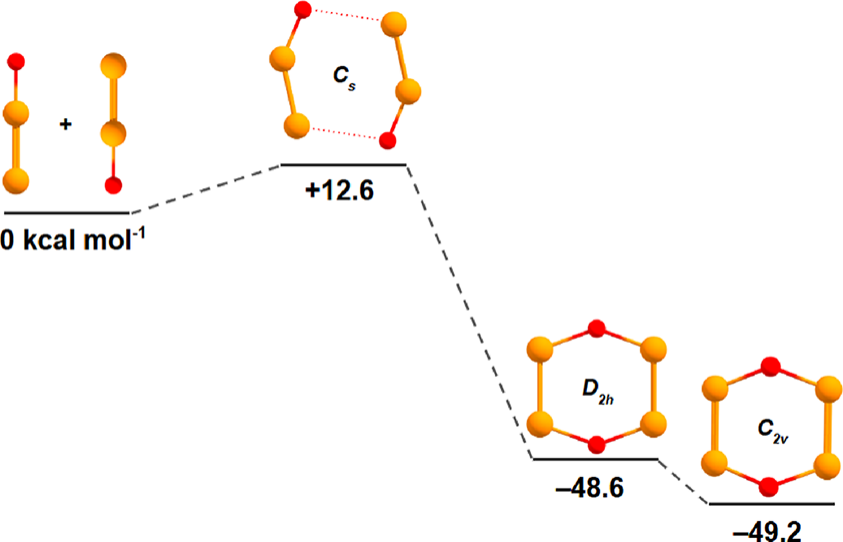
B3LYP/cc-pV­(T+d)­Z-optimized geometries with CCSD­(T)/cc-pV­(T+d)­Z single-point energy computations for stationary points along the 2P_2_O → cyclic P_4_O_2_ reaction coordinate.

It appears P_4_O_2_ species are formed from two distinct pathways in white phosphorus oxidation reactions. Clearly, the cyclic boat P_4_O_2_ structure is formed from the P_2_O dimerization reaction.^26^ More ambiguously, sequential addition of oxygen atoms to the P_4_ tetrahedron appears to form P_3_OPO and P_3_PO_2_. Andrews and co-workers proposed the reaction pathway P_4_ + O → T-P_4_O → P_3_–PO → B–P_4_O,^1,23^ where conversion of T-P_4_O to B–P_4_O goes through a P_3_–PO intermediate. The theoretical work by Yao and co-workers supports this claim,^39^ while theoretical results of Lohr suggest a single transition state connects the T- and B–P_4_O minima.^84^ If there is an intermediate P_3_–PO species, addition of an oxygen atom to the PO unit could yield our P_3_OPO and P_3_PO_2_ species. This is supported by the claim of Andrews and Mielke that two O_3_ molecules are required to produce these bands.^1^ The question of whether P_3_OPO and P_3_PO_2_ species are artifacts of matrix cages causing inordinate recombinations of P_3_ and PO_2_ units will remain undetermined until gas-phase studies are conducted.

### 3.3 Energies

Incremented focal-point energies, relative to tetrahedral P_4_ + ^3^Σ_*g*_^–^ O_2_, of the four P_4_O_2_ species are presented in Tables 5–8. *Bent*-P_3_OPO lies −93 kcal mol^–1^ lower in energy than ground-state P_4_ + O_2_, and was predicted to be the P_4_O_2_ global minimum by Peyerimhoff and co-workers.^38^ *Extended*-P_3_OPO and P_3_PO_2_ are found here to lie 5 and 8 kcal mol^–1^ higher in energy than *bent*-P_3_OPO, well under the energy available for formation under matrix conditions. Additional P_3_OPO conformers fall within this energy range, but were not investigated. All four P_4_O_2_ species have positive ZPVE corrections relative to the P_4_ + O_2_ ZPVE. This is counterintuitive when considering the strained tetrahedral P_4_ geometry, but the unbound P_4_ + O_2_ system as a whole would be less constrained than a bound P_4_O_2_ molecule. Further, five additional vibrational modes are present in P_4_O_2_ compared to the total number of modes in P_4_ + O_2_. Hence, positive ZPVE corrections for P_4_O_2_ species relative to P_4_ + O_2_ should be expected.

**Table 5:** Incremented Focal-Point Energies in kcal mol^–1^ for *Bent*-P_3_OPO^a^

*bent*-P_3_OPO							
total = −94.22 + 1.64 = −93 kcal mol^–1^							
	ΔHF	+ δ MP2	+ δ CCSD	+ δ CCSD(T)	+ δ CCSDT^b^	+ δ CCSDT(Q)^c^	net
cc-pV(D+d)Z	–76.82	+13.30	–10.84	+0.36	–0.66	+0.27	[−74.38]
cc-pV(T+d)Z	–93.58	+15.76	–12.95	+1.21	[−0.66]	[+0.27]	[−89.95]
cc-pV(Q+d)Z	–95.77	+14.76	–13.26	+1.36	[−0.66]	[+0.27]	[−93.30]
extrapolation	[−95.85]	[+14.02]	[−13.48]	[+1.47]	[−0.66]	[+0.27]	[−94.22]

a Total energies are relative to tetrahedral P_4_ + ^3^Σ_*g*_^–^ O_2_ and are reported as Net/Extrapolation + ΔZPVE.

b DLPNO–CCSDT – CCSD­(T).

c DLPNO–CCSDT­(Q) – DLPNO–CCSDT.

**6:** Incremented Focal-Point Energies in kcal mol^–1^ for *Extended*-P_3_OPO^a^

*extended*-P_3_OPO							
total = −89.88 + 1.81 = −88 kcal mol^–1^							
	ΔHF	+ δ MP2	+ δ CCSD	+ δ CCSD(T)	+ δ CCSDT^b^	+ δ CCSDT(Q)^c^	net
cc-pV(D+d)Z	–71.48	+13.23	–10.94	+0.50	–0.74	+0.29	[−69.13]
cc-pV(T+d)Z	–89.35	+15.92	–13.14	+1.29	[−0.74]	[+0.29]	[−85.72]
cc-pV(Q+d)Z	–91.79	+15.16	–13.47	+1.47	[−0.74]	[+0.29]	[−89.07]
extrapolation	[−91.93]	[+14.60]	[−13.71]	[+1.60]	[−0.74]	[+0.29]	[−89.88]

a Total energies are relative to tetrahedral P_4_ + ^3^Σ_*g*_^–^ O_2_ and are reported as Net/Extrapolation + ΔZPVE.

b DLPNO–CCSDT – CCSD­(T).

c DLPNO–CCSDT­(Q) – DLPNO–CCSDT.

**7:** Incremented Focal-Point Energies in kcal mol^–1^ for P_3_PO_2_ ^a^

P_3_PO_2_							
total = −86.28 + 1.27 = −85 kcal mol^–1^							
	ΔHF	+ δ MP2	+ δ CCSD	+ δ CCSD(T)	+ δ CCSDT^b^	+ δ CCSDT(Q)^c^	net
cc-pV(D+d)Z	–62.42	+4.99	–6.37	–0.95	–0.67	+0.02	[−65.40]
cc-pV(T+d)Z	–79.97	+7.68	–8.45	–0.38	[−0.67]	[+0.02]	[−81.78]
cc-pV(Q+d)Z	–81.91	+6.30	–8.73	–0.28	[−0.67]	[+0.02]	[−85.27]
extrapolation	[−81.79]	[+5.30]	[−8.94]	[−0.20]	[−0.67]	[+0.02]	[−86.28]

a Total energies are relative to tetrahedral P_4_ + ^3^Σ_*g*_^–^ O_2_ and are reported as Net/Extrapolation + ΔZPVE.

b DLPNO–CCSDT – CCSD­(T).

c DLPNO–CCSDT­(Q) – DLPNO–CCSDT.

**Table 8:** Incremented Focal-Point Energies in kcal mol^–1^ for *Cyclic*-P_4_O_2_ ^a^

*cyclic*-P_4_O_2_							
total = −64.36 + 1.66 = −63 kcal mol^–1^							
	ΔHF	+ δ MP2	+ δ CCSD	+ δ CCSD(T)	+ δ CCSDT^b^	+ δ CCSDT(Q)^c^	net
cc-pV(D+d)Z	–47.16	+23.30	–18.24	+0.79	–0.98	+0.31	[−41.98]
cc-pV(T+d)Z	–64.92	+26.63	–21.33	+1.91	[−0.98]	[+0.31]	[−58.39]
cc-pV(Q+d)Z	–68.43	+25.87	–21.82	+2.20	[−0.98]	[+0.31]	[−62.84]
extrapolation	[−69.25]	[+25.31]	[−22.17]	[+2.42]	[−0.98]	[+0.31]	[−64.36]

a Total energies are relative to tetrahedral P_4_ + ^3^Σ_*g*_^–^ O_2_ and are reported as Net/Extrapolation + ΔZPVE.

b DLPNO–CCSDT – CCSD­(T).

c DLPNO–CCSDT­(Q) – DLPNO–CCSDT.

Double-ζ basis sets are shown here to be inadequate for predicting energies of these P_4_O_2_ species, even if significant dynamic correlation is included. HF/DZ overestimates the energies of P_4_O_2_ species ca. 20 kcal mol^–1^, whereas HF/TZ predicts energies within 5 kcal mol^–1^ of the net extrapolation. MP2 raises the energy of the P_4_O_2_ species relative to P_4_ + O_2_, and CCSD lowers the energy so that the net impact of MP2 + CCSD on the total is at most a few kcal mol^–1^ no matter the degree of polarization of the basis set. If high-accuracy at the smallest computational cost is desired, DLPNO–CCSD­(T) with a triple-ζ basis set would be the best option available. Although archaic in comparison, and not recommended, HF/QZ may suffice for predicting energies of phosphorus oxides due to fortuitous error cancellations.

## 4 Conclusions

We present the first evidence for the formation of P_3_OPO and P_3_PO_2_ species from white phosphorus oxidation reactions based on our theoretical predictions and previous experimental work. Additional theoretical work is required to fully understand the P_4_O_*x*_ (*x* = 1–5) intermediates involved in white phosphorus oxidation pathways leading to P_4_O_6_ and P_4_O_10_. Experimental bands at 898 and 891 cm^–1^ were correctly assigned to P_4_O_2_ species,^1^ but peaks in the terminal P=O and −PO_2_ regions thought to solely belong to P_2_O_4_ species should be re-examined experimentally considering the peculiar geometries of our P_4_O_2_ molecules. Gas-phase direct absorption experiments may be sufficient to determine whether P_4_O_2_, P_2_O_4_, or a multitude of species are responsible for the −PO_2_ vibrational bands observed by Andrews and co-workers.^1,14^ However, computational work on charged phosphorus oxides is still encouraged, as ignition conditions encourage ion generation and action spectroscopy of mass-selected species would be suited to resolve −PO_2_ vibrational frequencies of charged P_4_O_2_ and P_2_O_4_ molecules separately.^89,90^ Full conformational sampling of the P_3_OPO and P_3_PO_2_ potential energy surfaces may also be necessary to determine the number of P_4_O_2_ bands expected in a given experiment. Cyclic P_4_O_2_ appears only in experiments with ample P_2_O production, suggesting P_2_O dimerization forms cyclic P_4_O_2_. P_3_OPO and P_3_PO_2_ form in experiments with sufficient P_4_ concentration, indicating they are formed from sequential oxygen additions to the P_4_ tetrahedron.
